# Bioaccumulation, Biosedimentation, and Health Hazards of Elements in Crayfish, *Procambarus clarkii* from El-Rahawi Drain and El-Qanatir in the River Nile, Egypt

**DOI:** 10.1007/s12011-022-03380-7

**Published:** 2022-08-19

**Authors:** Mahmoud Mahrous M. Abbas, Mohamed A.E. Abd El-Aziz, Mohamed M.Y. Kaddah, Abdel-Kader Hassan, Hussein A. El-Naggar, Mahmoud Radwan, Mohamed A.M. El-Tabakh, Moharam A. Afifi, Mansour A.E. Bashar

**Affiliations:** 1 Marine Biology Branch, Zoology Department, Faculty of Science, Al-Azhar University, Nasr City, 11884 Cairo Egypt; 2 Pharmaceutical and Fermentation Industries Development Center, City of Scientific Research and Technological Applications, New Borg El-Arab City, Alexandria, Egypt

**Keywords:** Elements, Lithium, El-Rahawi drain, Non-carcinogenic and carcinogenic risks, Sediment, Water

## Abstract

Elements accumulation in crayfish is proportional to the increase in bioavailability (direct contact) with the surrounding water, sediment, and feeding. Five heavy metals (Cu, Cr, Mn, Ni, and Ag) and lithium (Li) were analyzed in the sediment, water, and crayfish tissues. Elements (heavy metals and lithium) concentrations in sediment, water, and crayfish tissues showed significant differences between the two sampling stations (El-Qanatir and El-Rahawi drain). However, the levels of elements in crayfish tissues were arranged in declining order as hepatopancreas > gills > exoskeleton > muscles for Cu and Cr; hepatopancreas > exoskeleton > gills > muscles for Ni and Ag; and exoskeleton > gills > hepatopancreas > muscles for Li and Mn. The human health hazard evaluation of heavy metals and lithium exposure via edible tissue consumption was assessed for both children and adult consumers. The target hazard quotient THQ values of crayfish edible tissues (less than 1) will not impose any health implications for consumers who ingest edible tissues in sufficient quantities. Furthermore, the hazard index (HI) values reported for children and adult consumers were lower than one, indicating non-carcinogenic and carcinogenic hazards, suggesting that crayfish edible tissues are safe for human ingestion. This evidence also found that *Procambarus clarkii* could be a good bio-indicator organism for monitoring potentially metals in aquatic systems.

## Introduction

The Nile River basin is one of the most significant features of Africa’s northeastern basin section, extending for about 6825 km. In the delta, the Nile River is divided into two branches: Rosetta and Damietta. The Rosetta branch receives a variety of pollution categories from many sources, including sewage, domestic, industrial, and agricultural waste effluents from El-Rahawai drains, which total more than 5 × 10^8^ m^3^ per day [1, 2].

HMs pollution is becoming more of a hazard worldwide, particularly in Egypt. HMs levels widely absorbed and accumulated in tissue; they pose a risk to human health when ingested through contaminated food [3]. HMs levels are mainly correlated with sediments compared to water layers due to the presence of a variety of various, i.e., organic compounds, clay minerals, and metal oxides in the sediment layers [4]. Aquatic species accumulate trace metals in minor amounts via absorption from the water column and in higher quantities via biomagnification from their prey, whereas in humans, they might be ingested through the food chain, leading to acute and chronic health impacts [5]. Among aquatic species, the freshwater crayfish, ***Procambarus clarkii,*** receives HMs from the sediments and water in which it lives. Metal accumulation in crayfish tissues has been observed in various scientific studies [6, 7]. Apart from that, exogenous and endogenous variables regulate the bioaccumulation of metals in aquatic species. Environmental parameters such as metal bioactivity, temperatures, and alkalinity of ambient aquatic habitats reflect exogenous variables, whereas endogenous variables include lifetime, environment, size, gender, ecology, physiological operation, and feeding habits [8]. The bio-sedimentation factor is defined as the ratio of heavy metal concentration in the body of organisms to that in the sediment. It makes it possible to evaluate the effectiveness of the bioaccumulation of heavy metals in the organism and give an idea of the speed of substance absorption and excretion by a living organism [9, 10]. Pollutants can pose a significant risk to aquatic fauna through exposed, bioaccumulation, and biomagnification processes [11].

Potential health hazards related to elements exposure have been recognized in recent decades. According to recent studies [12], elements are either carcinogenic or non-carcinogenic to humans. According to their indecomposable and protracted nature inside the visceral organ categories of consumers, HMs pose health risks such as kidney failure, skeletal deformation, and liver failure [13]. As a result, it is critical to examine the potential risks to human health linked to the ingestion of contaminated food. The goals of this study were: (1) to compare heavy metals and lithium concentrations in crayfish tissues, sediment, and water from El-Qanatir and El-Rahawi stations; (2) to determine the bioaccumulation and biosedimentation factors of heavy metals and lithium; and (3) to evaluate the possible human health hazards associated with ingestion of crayfish muscle.

## Materials and Methods

### Collection of Water, Sediment, and Crayfish Samples

**Fig. 1:**
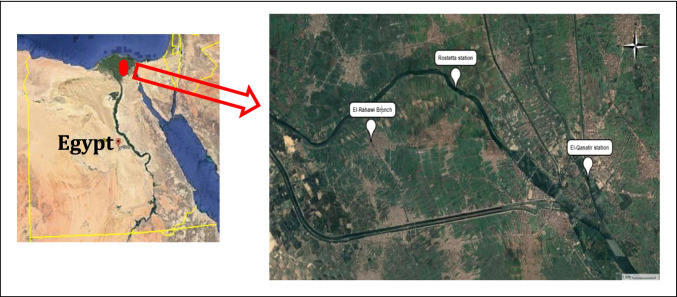
A map of the study stations showed El-Qanatir station and El-Rahawi drain

Sediment and water samples were collected from two locations: the first is upstream (El-Qanatir station) on the Nile River, and the second is the El-Rahawi drain, which discharges into the Nile near Rosetta Branch in El-Qalyubia Province (Fig. 1). Specimens were taken between the spring and summer of 2021. Fifty (50) crayfish, ***Procambarus clarkii,*** individuals were collected from each studied location by fisherman. Crayfish, *P. clarkii*, samples were obtained at the same time as water and sediment samples and transported to the laboratory of Environmental Physiology, Faculty of Science, Al-Azhar University, Egypt.

In the lab, crayfish were identified. Crayfish samples (individuals’ lengths ranged from 8.2 to 12.85 cm, with an average of 10.33 ± 1.69 cm, while their total weights ranged from 23.18 to 47.86 g, with an average of 38.52 ± 4.67 g) were re-washed carefully with potable water, then beheaded, skinned, and dissected (using plastic tools) to obtain exoskeleton, muscles, hepatopancreas, and gills for elements analysis.

### Elements Levels Measurement

Elements (manganese, Mn; copper, Cu; lithium, Li; nickel, Ni; chromium, Cr; and silver, Ag) were measured in water, sediment, and crayfish tissues (exoskeleton, muscles, hepatopancreas, and gills; *n* = 5) Heavy metals and lithium were determined in water samples using an acid digestion procedure according to [14]. However, sediment samples were air-dried, sieved (100 mesh). In a 50-mL digesting container, processed samples (0.5 g) were digested with HNO_3_ (65%, 5 mL), HF (40%, 2 mL), and HClO_4_ (40%, 1 mL) [14]. Furthermore, 0.5 g of crayfish tissues was dried, then digested in a flask with 2 mL ultrapure HNO_3_ (65%) and 1 mL H_2_O_2_ (30%). The mixture was heated to remove acid until the solutions were completely digested [15].

Finally, the digested mixtures of water, sediment, and tissues were transferred to a volumetric flask and diluted with de-ionized water. An inductively coupled plasma optical emission spectrophotometer (ICP-OES, Model 4300 DV, Perkin Elmer, Shelton, CT, USA) was used to measure the heavy metals and lithium in diluted water, tissues, and sediment solutions (*n* = 5). To estimate the ppm of each analyst in the diluted solution, samples were treated to a multi-element standard curve. The concentrations of elements in the water were reported in µg/L, whereas those in the crayfish tissues and sediment were expressed in µg/g on a dry weight basis.

### Bioaconcentration Factor Calculation

The bioaccumulation factor (Bio-AF) was calculated using the following formula [16] as: Bio-AF = C-crayfish / C-water, where C-crayfish is the concentration of elements in crayfish tissues (µg/kg) and C-water is the concentration of elements in water (µg/L). However, the biosedimentation factor (Bio-SF) was calculated using the following formula [17] as: Bio-SF = C-crayfish / C-sediment, where, C-crayfish, the concentration of elements in crayfish tissues (µg/g), and C-sediment, the concentration of elements in sediments (µg/g).

### Health Risk Assessment

The estimated daily intake (EDI) was computed using the formula below in mg^−1^ kg^−1^ day^−1^ units [18] as: EDI = (CF × IR × ER × EP / BW × AT) × 10^−3^, where CF stands for the elements concentration in crayfish muscle (mg/kg); IR is the intake rate of crayfish ingested (kg/day), which in this investigation was 7.9 g/day for children and 20.1 g/day for adults; ER stands for the exposure rate (365 days/year); EP stands for the exposure time over a lifetime (assumed to be 70 years old); BW stands for body weight, which is 70 kg for adults and 52.5 kg for children 6–11 years old, which is the 95^th^ percentile [19]; AT stands for an average lifetime (70 years, 365 days/year). However, the formula below, which was developed from the ratio of EDI to RfD (oral reference dose of HMs [20], was used to determine target hazard quotient (THQ) as: THQ = EDI / RfD, where RfD stands for elements oral reference doses (mg/kg/days). Moreover, the hazard index (HI) is a mathematical formula that calculates the non-carcinogenic hazard by summing the THQ values of metals under study [20] as follows: HI = THQ(Mn) + THQ(Cu) + THQ(Li) + THQ(Ni) + THQ(Cr) + THQ(Ag). Furthermore, the incremental likelihood of an individual developing cancer depends on the cancer slope factor (CSF), which is defined as the lifelong CR of exposure to HMs. The following formula was used to calculate the carcinogenic risk (CR) as: CR = (ER × EP × EDI × CSF × 10^–3^) / AT [21], where CSF refers to carcinogenic slope factor, and the CSF values for Ni and Cr are 8.4E − 4 and 4.1E − 2 mg kg^−1^ day^−1^, respectively [22].

### Statistical Analysis

The statistical analyses were performed using IBM SPSS Statistics Version 22; SPSS Inc., IL, USA, and when significant differences of one-way analysis of variance (ANOVA) were found, multivariate, post hoc Tukey evaluations were utilized to quantify the statistical difference between the elements levels in various crayfish tissues for each element. On the other hand, the independent-samples *t* test was used to investigate the statistical differences between the two locations (El-Qanatir and El-Rahawi drain) based on elements levels in water and sediments. The Pearson’s correlation coefficient between elements levels in the sediment, water, and studied crayfish tissues was investigated. However, statistical significance was performed at *p* < 0.05.

## Results and Discussion

### Elements Levels in Water and Sediment Samples

Table 1 shows the levels of heavy metals and lithium in the water of the study stations (El-Qanatir and El-Rahawi stations). Except for silver, the levels of elements (Mn, Cu, Li, Ni, and Cr) in the water of the examined stations were significantly higher in El-Rahawi drain compared to El-Qanatir station (*t* test, *p* < 0.05). However, the highest value of elements (54.55 ± 7.00 µg/L) was recorded in the El-Rahawi drain station for copper, and the lowest value (0.1 ± 0.005 µg/L) was recorded in both stations for silver, with elements arranged as Cu > Mn > Li > Ni > Cr > Ag in the El-Rahawi drain, and Mn > Cu > Li > Ni > Cr > Ag in the El-Qanatir station.

**Table 1:** Concentrations of heavy metals and lithium (mean ± SD, *n* = 5) in the water and sediment from El-Qanatir and El-Rahawi stations

	Water samples **(**µg/L)			[24]	Sediment samples (µg/g dw)			[31]
	El-Qanatir	El-Rahawi drain	Sig.*		El-Qanatir	El-Rahawi drain	Sig.*	
Cr	0.59 ± 0.14	1.19 ± 0.27	0.027	50	3.70 ± 0.65	23.24 ± 1.79	< 0.0001	5
Cu	12.20 ± 0.93	54.55 ± 7.00	0.0005	2000	12.98 ± 1.73	29.89 ± 2.65	0.004	40
Ni	0.69 ± 0.14	2.56 ± 0.53	0.0002	70	6.26 ± 1.07	31.85 ± 2.77	0.001	–
Mn	14.86 ± 1.82	20.61 ± 1.39	0.004	400	78.05 ± 5.16	255.69 ± 8.50	0.008	500
Li	2.30 ± 0.29	7.66 ± 0.66	0.012	–	28.06 ± 1.33	87.28 ± 2.52	0.008	–
Ag	0.1 ± 0.005	0.1 ± 0.005	0.067	–	1.65 ± 0.68	3.52 ± 0.14	0.093	–

^*^t test was used to detect the significant differences between two locations

In Egypt and other developing countries, where environmental protection laws have not been implemented, industrial and domestic wastes are dumped indiscriminately, entering water bodies. Toxic and hazardous chemicals, including metals, have been found in these wastes. Because of their toxicity, durability, and bioaccumulative nature, heavy metals poisoning of water resources is a major problem [23]. A comparison of the current study’s heavy metals levels in water and sediment with previous studies (Table 2). The Cr, Cu, Ni, and Mn levels in the water of the study stations were lower than WHO’s standard permissible values [24]. The level of Cr in the current study was lower than those recorded [25, 26]. The Cu level in the current study was within that mentioned [25, 27] . However, it was lower than that detected [28]. Moreover, it was higher than those recorded [26, 29]. The level of Ni in the current study was higher than that reported [29]. Moreover, it was lower than those determined [26–28]. The level of Mn in the current study was lower than that recorded by [25–29].

**Table 2:** Comparison between heavy metals levels in water and sediment of the current study with the previous studies

	Cr	Cu	Mn	Ni	Area
Water (μg/L)	0.59–1.19	12.2–54.55	14.86–20.61	0.69–2.56	Current study
[28] mg/L	––	0.05 ± 0.01	0.16 ± 0.01	0.03 ± 0.00	Rosetta branch
[25] mg/L	0.088 ± 0.16	0.054 ± 0.03	0.099 ± 0.15	––	Rosetta branch
[27] mg/L	––	0.006–0.01	0.044–0.064	0.001–0.003	River Nile
[26] mg/L	0.061 ± 0.06	0.009 ± 0.005	0.105 ± 0.087	0.017 ± 0.017	River Nile
[29] μg/L	––	1.14 ± 2.89	28.29 ± 19.98	0.89 ± 1.44	Rosetta branch
Sediment (µg/g dw)	166.87	13.47	21.44	19.06	**Current study**
[32]	37.1–233.1	11.8–60.20	125–1008.2	< 0.005	
[28]	––	4.1 ± 0.17	17.11 ± 0.27	2.38 ± 0.11	Rosetta branch
[33]	13.2–210	10.40	1287.5	15.57	**River Nile**

Sediment pollution is one of the most serious environmental issues affecting ecosystems, as sediments serve as both sinks and sources of toxins in aquatic systems. Sediment analysis is crucial for determining the degree of pollution in the environment [30]. Result reported that El-Rahawi drain station showed significantly higher levels of heavy metals and lithium in the sediment than El-Qanatir station (***t*** test***, p*** < 0.05, Table 1). The highest value of elements (255.69 ± 8.50 µg/g dw) was recorded in the El-Rahawi drain for manganese, and the lowest value (1.65 ± 0.68 µg/g dw) was noted in the El-Qanatir stations for silver, with elements arranged in the El-Rahawi drain as Mn > Li > Ni > Cu > Cr > Ag and in the El-Qanatir station as Mn > Li > Cu > Ni > Cr.

The concentrations of copper and manganese in sediment of studied stations were lower than the standard acceptable values, according to [31]. However, the Cr level in the El-Rahawi drain sediment was above the acceptable limit [31]. Levels of Cr in the current study were within those recorded [32, 33]. The levels of Cu in the current study were within range of those detected [32]. However, it was higher than those recorded [28, 33]. Levels of Ni in the current study were higher than those reported [28, 32, 33]. Levels of Mn in the current study were higher than those determined [28]. Moreover, it was lower than those recorded [32, 33].

### Elements Levels in Crayfish Tissues

Elements are accumulated by freshwater *P. clarkii*, crayfish in their tissues from the water, and sediments in which they live. Environmental factors affect metal bioaccumulation in aquatic species, but so do internal factors, including life cycle, habitat, and feeding habits [8]. The levels of heavy metals and lithium in the tissues of crayfish, *P. clarkii* collected from El-Qanatir and El-Rahawi stations, were represented in Table 3. The highest value of elements levels in the muscles was recorded in El-Rahawi drain for manganese (5.41 ± 0.08 µg/g dw), and the lowest value (0.24 ± 0.05 µg/g dw) was determined in El-Qanatir station for silver. However, the maximum values of elements levels in the crayfish exoskeleton collected from El-Rahawi drain were recorded for manganese (51.77 ± 2.08 µg/g dw), and the minimum values were determined in El-Qanatir station for chromium (1.20 ± 0.10 µg/g dw). Moreover, the maximal value of elements levels in the crayfish gills was recorded in El-Rahawi drain for manganese (63.40 ± 1.94 µg/g dw), while the minimal value (0.44 ± 0.02 µg/g dw) was determined in El-Qanatir station for silver. Furthermore, the highest value of elements levels in the hepatopancreas was recorded in El-Rahawi drain for copper (100.12 ± 3.21 µg/g dw), while the lowest value (3.29 ± 0.91 µg/g dw) was determined in the El-Qanatir station for lithium. There were significant differences (***p*** **<** 0.05) in the one-way analysis of variance between the different stations and organs, for all metals. Table 4 shows the Pearson correlation based on values of heavy metals and lithium in water, sediment, and tissues of crayfish collected from El-Qanatir and El-Rahawi drain stations. El-Rahawi drain had significantly higher levels of elements in water, sediment, and crayfish tissues than El-Qanatir station (***t*** test***, p*** < 0.05), which may be due to the increased influence of sewage, industrial, and agricultural discharge, as well as deposition of these metals from the atmosphere. This finding agrees with [23, 34]

**Table 3:** Heavy metals and lithium levels (mean ± SD, µg/g dw, *n* = 5) in crayfish, *Procambarus clarkii* collected from El-Qanatir and El-Rahawi stations

		Muscles	Exoskeleton	Gills	Hepatopancreas
Cr	El-Qanatir	0.38 ± 0.03 dB	1.20 ± 0.10 ^cB^	1.74 ± 0.23 ^bB^	14.34 ± 1.98 ^aB^
	El-Rahawi drain	0.42 ± 0.02 ^dA^	1.32 ± 0.18 ^cA^	1.87 ± 0.24 ^bA^	19.85 ± 2.98 ^aA^
Cu	El-Qanatir	3.81 ± 0.34 dB	8.27 ± 0.39 ^cB^	32.25 ± 1.06 ^bB^	80.24 ± 2.21 ^aB^
	El-Rahawi drain	4.24 ± 0.11 ^dA^	8.69 ± 0.74 ^cA^	47.71 ± 1.67 ^bA^	100.12 ± 3.21 ^aA^
Ni	El-Qanatir	0.40 ± 0.05 dB	2.91 ± 0.37 ^bB^	1.86 ± 0.22 ^cB^	4.91 ± 0.17 ^aB^
	El-Rahawi drain	0.50 ± 0.01 ^dA^	3.03 ± 0.37 ^bA^	2.15 ± 0.24 ^cA^	5.34 ± 0.18 ^aA^
Mn	El-Qanatir	4.36 ± 0.94 dB	53.89 ± 3.47 ^aB^	33.71 ± 3.59 ^bB^	17.21 ± 0.36 ^cB^
	El-Rahawi drain	5.41 ± 0.08 ^dA^	63.40 ± 1.94 ^aA^	51.77 ± 2.08 ^bA^	23.37 ± 1.94 ^cA^
Li	El-Qanatir	1.09 ± 0.03 d B	4.66 ± 0.23 ^aB^	4.09 ± 0.89 ^bB^	3.29 ± 0.91 ^cB^
	El-Rahawi drain	1.22 ± 0.01 ^dA^	4.87 ± 0.17 ^aA^	4.18 ± 0.68 ^bA^	3.16 ± 0.48 ^cA^
Ag	El-Qanatir	0.24 ± 0.05 dB	2.21 ± 0.13 ^bB^	0.44 ± 0.02 ^cB^	4.60 ± 0.84 ^aB^
	El-Rahawi drain	0.37 ± 0.09 ^dA^	2.36 ± 0.23 ^bA^	0.57 ± 0.01 ^cA^	5.13 ± 0.67 ^aA^

The outcomes from the same row and location with different small alphabetic letters differ statistically (ANOVA, *p* < 0.05). On the other hand, different superscript with capital letters in the same column and tissue for each metal are significantly different (*t* test, *p* < 0.05)

**Table 4:** Pearson c orrelation coefficients b ased on heavy metals and lithium levels in the water, sediment, and tissues of crayfish from the study stations. Values with numeric bold is significant at *p* < 0.05

*HM*		El-Rahawi drain					El-Qanatir				
		*Cr*	*Cu*	*Li*	*Ag*	*Mn*	*Cr*	*Cu*	*Li*	*Ag*	*Mn*
***Water***	Cu	− 0.42	1				− **0.93**	1			
	Li	**0.85**	0.12	1			− 0.23	**0.57**	1		
	Ag	0.32	**0.73**	**0.77**	1		− **0.94**	**0.89**	**0.54**	1	
	Mn	**0.72**	0.33	**0.98**	**0.89**	1	**0.65**	− **0.88**	− **0.89**	− **0.87**	1
	Ni	**0.85**	− 0.42	**0.85**	0.32	**0.72**	**0.79**	− **0.93**	− 0.23	− **0.94**	**0.65**
Sediment	Cu	0.07	1				− **0.71**	1			
	Li	**0.71**	0.75	1			− 0.28	**0.87**	1		
	Ag	**0.97**	0.32	**0.87**	1		**0.67**	0.04	**0.53**	1	
	Mn	0.28	− **0.87**	− **0.70**	− 0.25	1	**0.73**	− **0.68**	− 0.23	**0.71**	1
	Ni	**0.77**	**0.69**	**0.86**	**0.91**	− **0.63**	− **0.52**	− 0.23	− **0.68**	− **0.98**	− **0.56**
Tissues	Cu	**0.89**	1				**0.91**	1			
	Li	**0.90**	**0.79**	1			**0.89**	**0.81**	1		
	Ag	− 0.04	0.11	0.13	1		− 0.06	0.05	0.14	1	
	Mn	− 0.20	− 0.05	− 0.03	**0.94**	1	− 0.23	− 0.16	− 0.11	**0.85**	1
	Ni	**0.80 **	**0.81 **	**0.93**	0.40	0.25	**0.80**	**0.79**	**0.94**	0.41	0.16

 The presented data reports that one-way analysis of variance (ANOVA) based on heavy metals and lithium in the different tissues of crayfish revealed significant differences between crayfish tissues in both studied locations (ANOVA*, p* < 0.05). Additionally, heavy metals and lithium in the different tissues of crayfish were ordered in descending order according to; hepatopancreas > gills > exoskeleton > muscles for Cu and Cr; hepatopancreas > exoskeleton > gills > muscles for Ni and Ag; exoskeleton > gills > hepatopancreas > muscles for Li and Mn. This finding agrees with [7], who mentioned that the bio-accumulation of Cu in the tissues of *Procambarus clarkii* was hepatopancreas > gills > exoskeleton > muscles. However, Mn was ordered in the following order: exoskeleton > gills > hepatopancreas > muscles of crayfish. Manganese is in direct contact with the crayfish shell, and its level in the shell was higher than in water, due to its assimilation into the calcium carbonate structure [7].

**Fig. 2:**
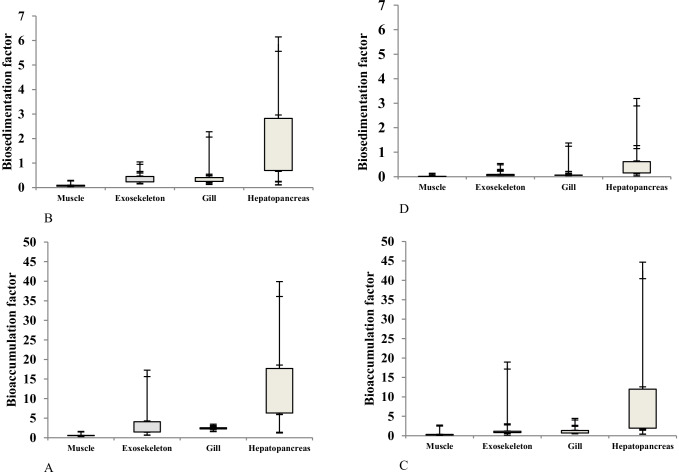
Box-plot distributions of Bio-AF and Bio-SF of heavy metals and lithium in the tissues of crayfish; **A** is Bio-AF; **B** is Bio-SF for El-Qanatir station; **C** is Bio-AF; and **D** is Bio-SF for El-Rahawi drain

**Fig. 3:**
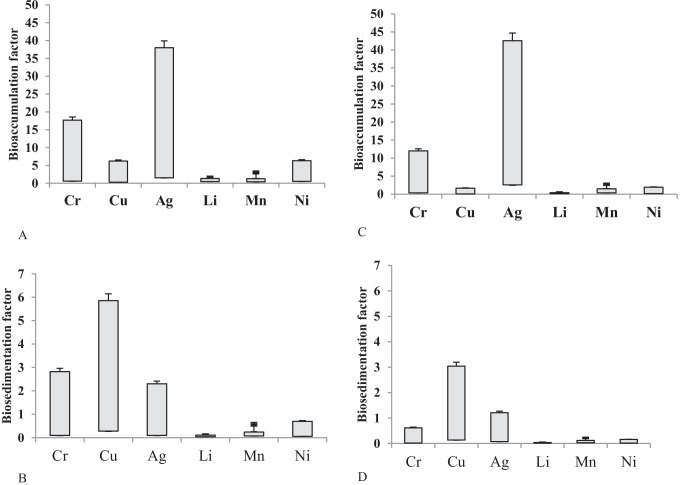
Box-plot distributions of Bio-AF and Bio-SF of heavy metals and lithium in the tissues of crayfish; **A** is Bio-AF; **B** is Bio-SF for El-Qanatir station; **C** is Bio-AF; and **D** is Bio-SF for El-Rahawi drain

The highest values of Bio-AF and Bio-SF of heavy metals and lithium in the tissues of *P. clarkii* crayfish taken from El-Qanatir and El-Rahawi stations were recorded in hepatopancreas and the lowest observed in the muscles (Figs. 2–3).The high metal accumulation in the crayfish hepatopancreas may explain the synthesis of low-molecular-weight metal-binding (metallothionein-like) proteins, which have been widely recorded in different crustacean species [35]. On the other hand, the metal concentrations were accumulated in the muscles of crayfish at the lowest values [22]. Many authors have noted a similar accumulation pattern for various HMs, indicating that higher accumulations were observed in the exoskeleton, gills, and hepatopancreas, whereas lower accumulations were observed in the muscle tissues [6, 7]. Each metal exhibits a different accumulation pattern based on its interaction with crayfish metabolism. Non-essential metals have no metabolic function in crustacean species, and the tissue contents of these metals are unregulated [35].

The levels of Li and Ag in crayfish tissues can thus be used to estimate metal levels in the environment. Thus, the levels of Li and Ag in *P. clarkii* reflect the levels of these metals in the water and sediment. Cu and Ni are also widely found in the hepatopancreas of other crayfish species [6]. Regardless of their environmental levels, Cu is found in high concentrations in crayfish tissues. Cu bioaccumulation in crayfish tissues increases significantly whenever their environmental content is increased [36].

The BIO-SF is the ratio of metal levels in the liver (hepatopancreas) to sediment levels [37]. Organisms are divided into three groups based on their BIO-SF values: (1) If the BIO-SF is greater than 2, the organism is a macro-concentrator. (2) If 1 < BIO-SF < 2, it indicates that the organism is a micro-concentrator. (3) If BIO-SF is lower than 1, the organism is de-concentrating the metal and releasing it into the sediment [38]. Based on the above, the crayfish tissues from El-Qanatir station showed that BIO-SF of muscles is de-concentrators for all metals and releases the metals in sediment. BIO-SF of the exoskeleton is de-concentrators for all metals except Ag. BIO-SF of gills is de-concentrators for all metals except Cu. BIO-SF of exoskeleton is de-concentrators for all metals except Ag.

### Human Health Risk Assessment

**Fig. 4:**
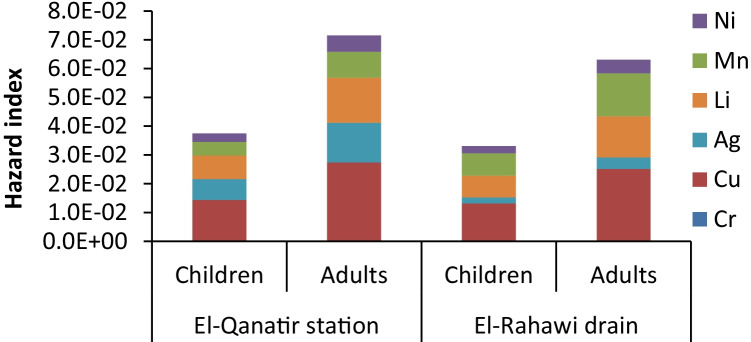
Values of hazard index (HI) of heavy metals and lithium in crayfish muscles from study stations

**Fig. 5:**
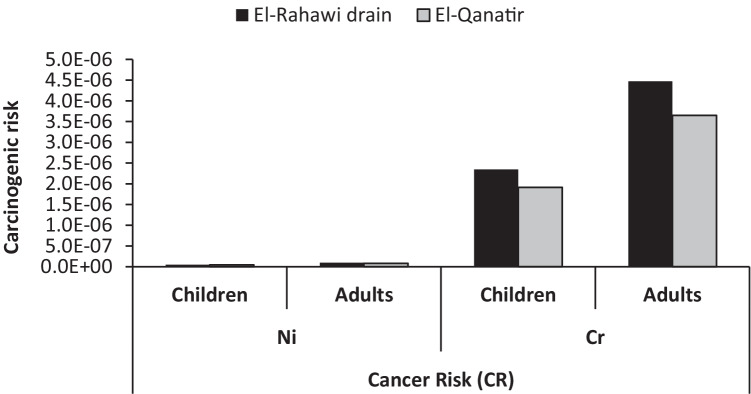
Carcinogenic risk (CR) of Ni and Cr from consumption of crayfish from study stations

The EDIs of the metal ions under investigation are lower than RfDs (oral reference dosages) [21, 22]. As a result, normal crayfish ingestion should not pose a serious health risk to consumers, while caution should be exercised to avoid excessive consumption. The estimated daily intake (EDI, mg/kg/day) and the target hazard quotients (THQ) for metals in crayfish muscles was shown in Table 5. The sequence of EDI values in crayfish from El-Qanatir stations, it was ranged from 3.61E − 05 mg/kg/day for children consumers to 1.25E − 03 mg/kg/day for adults. However, in El-Rahawi drain, it fluctuated between 1.05E − 05 mg/kg/day for children consumers and 2.08E − 03 mg/kg/day for adult consumers. The 95^th^ percentile of the THQ and HI was used to rank the non-carcinogenic risk of each metal among species and tissues [39]. The values of THQ in the studied stations were varied from 2.5E − 03 for children to 4.02E − 02 for adults. Additionally, the hazard index (HI) in the muscles of crayfish was 4.86E − 02 for the children’s consumer and 1.08E − 01 for the adult consumers (Fig. 4). THQs and HI calculated for heavy metals and lithium in crayfish are less than 1, which indicates that crayfish consumption does not pose a non-carcinogenic hazard for children and adults who consume crayfish in sufficient quantities. The CR values for Ni and Cr in crayfish muscles from the El-Rahawi drain and El-Qanatir stations were calculated for both children and adult consumers, and the findings are shown in Fig. 5. In both study locations, the CR values of Cr varied between 1.9E − 06 and 4.9E − 06. However, CR values of Ni in both study locations ranged from 4.2E − 08 to 1.2E − 07. Using the acceptable limit of E − 4, the carcinogenic risk (CR) values for Ni and Cr do not pose a carcinogenic risk to children and adult consumers of crayfish muscles in both studied stations as the CR values are less than 1E − 6 for both children and adult consumers [21, 22, 39].

**Table 5:** Values of EDI (mg/kg/day), THQ, and HI of heavy metals and lithium in the muscles of crayfish from the study stations

		El-Rahawi drain		El-Qanatir station		RfD [20]
		Children	Adults	Children	Adults	
EDI (mg/kg/day)	Cr	5.72E − 05	1.09E − 04	4.66E − 05	8.90E − 05	15E − 1
	Cu	5.73E − 04	1.09E − 03	5.25E − 04	1.00E − 03	40E − 3
	Ag	3.61E − 05	6.89E − 05	1.05E − 05	2.01E − 05	5E − 3
	Li	1.64E − 04	3.13E − 04	1.50E − 04	2.87E − 04	20E − 3
	Mn	6.56E − 04	1.25E − 03	1.09E − 03	2.08E − 03	14E − 2
	Ni	6.02E − 05	1.15E − 04	4.97E − 05	9.48E − 05	20E − 3
THQ	Cr	1.91E − 02	3.64E − 02	1.6E − 02	3.0E − 02	
	Cu	1.43E − 02	2.74E − 02	1.3E − 02	2.5E − 02	
	Ag	7.22E − 03	1.38E − 02	2.7E − 03	4.0E − 03	
	Li	8.20E − 03	1.56E − 02	7.5E − 03	1.4E − 02	
	Mn	4.69E − 03	8.94E − 03	7.8E − 03	1.5E − 02	
	Ni	3.01E − 03	5.74E − 03	2.5E − 03	4.7E − 03	
Hazard index (HI)		3.75E − 02	7.15E − 02	3.31E − 02	6.31E − 02

## Conclusion

Elements in water, sediment, and crayfish tissues were significantly higher in El-Rahawi drain than in El-Qanatir station. This indicates that El-Rahawi drain has more elements pollution than El-Qanatir station. The hepatopancreas of crayfish accumulated higher levels of elements than the other tissues, which is consistent with previous research. However, crayfish muscle accumulated elements at the lowest levels. Finally, the studied heavy metals and lithium will not cause any considerable adverse health effects to humans based on estimated daily intake, carcinogenic hazards, and non-carcinogenic hazards. Therefore, crayfish muscles are suitable for human consumption.

## Data Availability

The authors declare that data are available from the corresponding author at readers’ request.
